# Effects of sand burial depth on *Xanthium spinosum* seed germination and seedling growth

**DOI:** 10.1186/s12870-022-03424-z

**Published:** 2022-01-21

**Authors:** Yuan-yuan Tao, Tian-cui Shang, Jun-jie Yan, Yun-xia Hu, Yu Zhao, Ying Liu

**Affiliations:** 1 History, Geography and Tourism School, Shangrao Normal University, Shangrao, 334001 China; 2 Biology and Geography Science School, Yili Normal University, Yining, 835000 China

**Keywords:** *Xanthium spinosum*, Sand burial, Germination, Seedling growth, Biomass

## Abstract

In desert habitats, sand burial is an important factor affecting germination of plant seeds and seedling growth. *Xanthium spinosum* has strong adaptability in arid desert areas, and is a common malignant invasive plant in Xinjiang, China. The effects of different sand burial depths on seed germination, seedling emergence, growth and biomass allocation were studied to provide a scientific basis for further control of *X. spinosum*. Six sand burial depths (1, 2, 3, 5, 7 and 9 cm) were established to explore the response of *X. spinosum* seed germination and seedling growth to sand burial. The first emergence time, peak emergence time, emergence rate, seedling growth height, biomass and biomass distribution of *X. spinosum* seeds was significantly different at sand burial depths (*P* < 0.05). The *X. spinosum* seeds had the highest emergence rate (71.5%) at 1 cm sand burial and the maximum seedling height (7.1 cm). As sand burial depth increased, the emergence rate and seedling height gradually decreased. Emergence rate (12.25%) and seedling height (2.9 cm) were lowest at 9 cm sand burial. The root length at 9 cm depth (13.6 cm) was significantly higher than that at other sand depths (*P* < 0.05). The sand burial depth affected the biomass accumulation and distribution of *X. spinosum*. As sand burial depth increased, the root biomass and rhizome ratio increased, and the most deeply buried seedlings allocated more biomass for root growth. The optimal sand burial depth for seed germination and seedling growth of *X. spinosum* was 1–3 cm, and high burial depth (5–9 cm) was not conducive to the germination and growth of *X. spinosum* seedlings. For prevention and control of *X. spinosum*, we suggest deeply ploughing crops before sowing to ensure *X. spinosum* seeds are ploughed into a deep soil layer.

## Background

In the life cycle of higher plants, the process of seed germination and seedling establishment is the most vulnerable stage for settlement and population establishment; comprises the period of highest sensitivity to habitat conditions; and affects the population number, regeneration rate and community composition [21]. Seed germination is controlled by a mixture of its own internal factors and environmental factors [6]. The internal factors include seed quality, seed coat thickness, appendages and structure of the seed surface [23], while environmental factors include soil moisture, temperature, light, soil CO_2_ concentration and sowing or burial depth [3, 6, 29, 30]. In arid desert areas, because of the strong wind–sand interaction, sand burial is often the primary factor affecting germination [7]. It also affects plant reproduction and biomass allocation, and can even lead to plant death [10, 18, 23].

In desert habitats, different sand burial depths can produce selective pressures on seed germination and seedling morphology [26]. When the seeds are buried in shallow sand, they are subjected to lower temperature compared with that outside the soil, suitable light conditions, and a humid environment [11, 17, 29], which stimulate germination and growth. When the seed is buried too deep in the sand, the air permeability of the soil is reduced and the light is weakened, thereby inhibiting seed germination and seedling emergence [4, 8, 19]. Therefore, in-depth study of the impact of sand burial on plant seed emergence and seedling growth in desert areas has important theoretical and practical significance.

Among the Asteraceae plants, *Xanthium spinosum* (spiny cocklebur) is an herbaceous annual weed, a malignant weed that spreads widely in the world but is most widely distributed in Central and Southern Europe and the Pacific Northwest [2, 9]. In China, *X. spinosum* was first discovered in Dancheng County, Henan Province, and is now widely distributed in eastern Henan, northwest Anhui, Fengtai District in Beijing, Dalian in Liaoning, Hohhot in Inner Mongolia and Zhongwei in Ningxia [27]. In 2006, *X. spinosum* was first discovered in the Ili River Valley in Xinjiang, China [5]. Because the surface of the involucre is barbed, it is easily dispersed by people unintentionally, or spread on the fur of animals, and thus can invade a large area in a short time. As *X. spinosum* can adapt to the desert habitat in Xinjiang, China, a large area of invasive populations has formed in the Takermohuer Desert in the Yili River Valley, and the weed has rapidly invaded the oasis, grassland and villages surrounding the desert.

*X. spinosum* has strong adaptability in the desert, and often forms mono-dominant community. At the end of each growing season, a large number of mature involucres form a seed bank, which provides a continuous seed source for community succession, malignant expansion and invasion of sandy habitats, and further aggravates the invasion of the surrounding environment. At present, research on *X. spinosum* has mainly focused on the separation and identification of its active components, allelopathy of secondary metabolites, invasion and distribution, and interspecific competition [25]. To our knowledge, no studies have considered the influence of sand burial on the output, settlement, population establishment and community succession of the *X. spinosum* seed bank. Furthermore there has been no consideration of the influence of sand burial on seed germination, seedling growth and morphogenesis of *X. spinosum*. Therefore, this study examined the effects of different sand burial depths on *X. spinosum* seed germination, seedling emergence, growth and biomass allocation in the Yili River Valley. Based on experiments with different sand burial depths, this study aims to (1) determine whether different sand burial depths have any influence on the growth of *X. spinosum* seedlings; (2) explore how different sand burial depths affect the biomass distribution pattern of *X. spinosum*; and (3) reveal the relationship between seedling emergence, growth and sand burial depth, as well as examine the ecological adaptability of seed germination and seedling growth to sand burial.

## Results

### Effects of different sand burial depths on the emergence process of *X. spinosum* seeds

Different sand burial depths have substantial effects on the initial emergence time of *X. spinosum* and the peak time of emergence. At a sand depth of 1 cm, the initial emergence time was shortest at about 5 days, whereas at 9 cm sand burial depth, the time required for seedlings to be unearthed was longest at about 22 days. In the 3–9 cm treatment group, the deeper the sand burial depth, the longer the time needed for seedling emergence.

The emergence peak time was about 1–2 days after the first emergence time, and had a value of 1 day after the first emergence time when the sand burial depth was 1 cm, whereas the other treatment groups reached the emergence peak after 2 days (Table 1).

**Table 1:** Effect of sand burial depth on the emergence process of *X. spinosum* seeds

Sand burial depth (cm)	Seeding emergence initiation time (d)	Seeding emergence peak time (d)
1	5 ± 0.05d	6 ± 0.10d
2	8 ± 0.13ab	10 ± 0.47ab
3	6 ± 0.15c	8 ± 0.14c
5	8 ± 0.22b	10 ± 0.52b
7	11 ± 0.55a	13 ± 0.22a
9	22 ± 0.51a	22 ± 0.00a

The values were represented as means ± SE. Different lowercase letters indicate significant differences among the different sand burial depths of *X. spinosum* by using one-way ANOVA from LSD tests (*P* < 0.05)

**Fig. 1:**
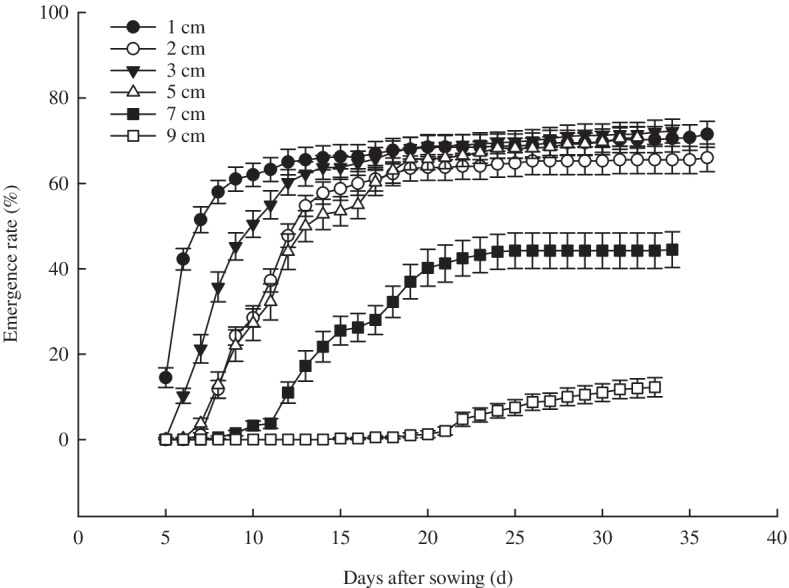
Effects of different sand burial depths on the emergence process of *X. spinosum* seeds. (The values were represented as means ± SE)

Figure 1 shows that the emergence rate of *X. spinosum* seeds decreased with the increase of sand burial depth. The emergence rate was fastest at 1 cm sand burial depth, with values of 14.5% and 62% on the 5 days and 10 days after sowing, respectively, and reached 68% after sowing for 20 days. Subsequently, the emergence rate only increased slowly, with the highest emergence rate value of 71.5%. The emergence of seedlings was slowest at 9 cm depth, and they emerged for the first time at 22 days after sowing, with an emergence rate of only 4.75%, and maximum of only 12.25%. The emergence rate of 7 cm depth was 3.75% at 11 days and 17.15% at 13 days, and the highest emergence rate was only 44.25%. There was no significant difference between the emergence rates of 1, 2, 3 and 5 cm after 20 days.

### Relationship between seedling height and sowing days at different sand burial depths

**Fig. 2:**
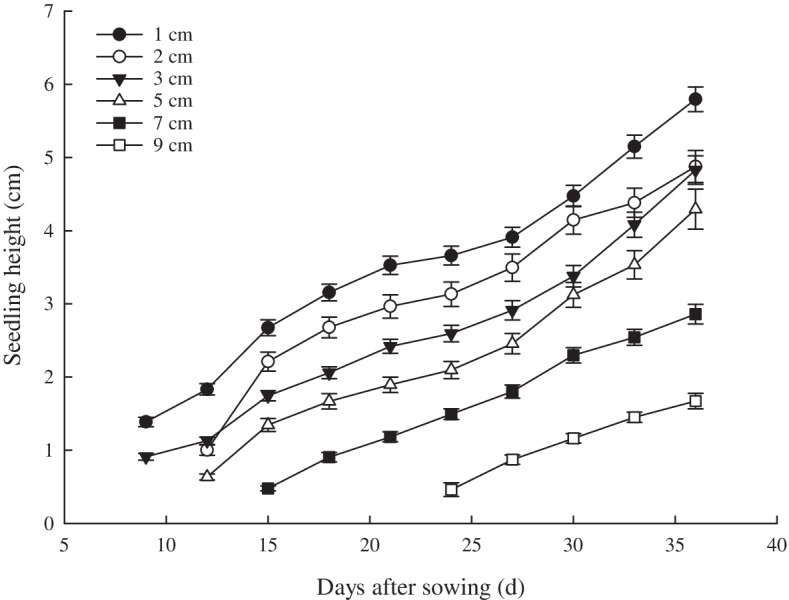
Relationship between seedling height and sowing days of *X. spinosum* at different sand burial depths. (The values were represented as means ± SE)

Figure 2 shows that the seedling height of *X. spinosum* was substantially affected by different sand burial depth. As sand burial depth increased, the seedling height gradually decreased. The seedling height at 1-cm burial depth was significantly higher than that at other sand burial depths (*P* < 0.05). From the 15th day, the seed height under 1-, 2-,3-,5- and 7-cm sand burial depth began to change significantly, and continued to the end of the experimental period. Compared with 1-, 2-,3- and 5-cm sand burial depth, the depth of 7 cm and 9 cm were significantly inhibited within 10-20 days after sowing. The heights at burial depths of 7 and 9 cm were not recorded until 15 and 24 days after sowing, respectively. At the end of the experiment, 36 days after sowing, the height of seedlings at 1 cm sand burial depth was the highest at 5.8 cm, and the height of seedlings at 9 cm sand burial depth was the lowest at 1.67 cm.

### Morphological characteristics of *X. spinosum* seedlings at different sand burial depths

**Fig. 3:**
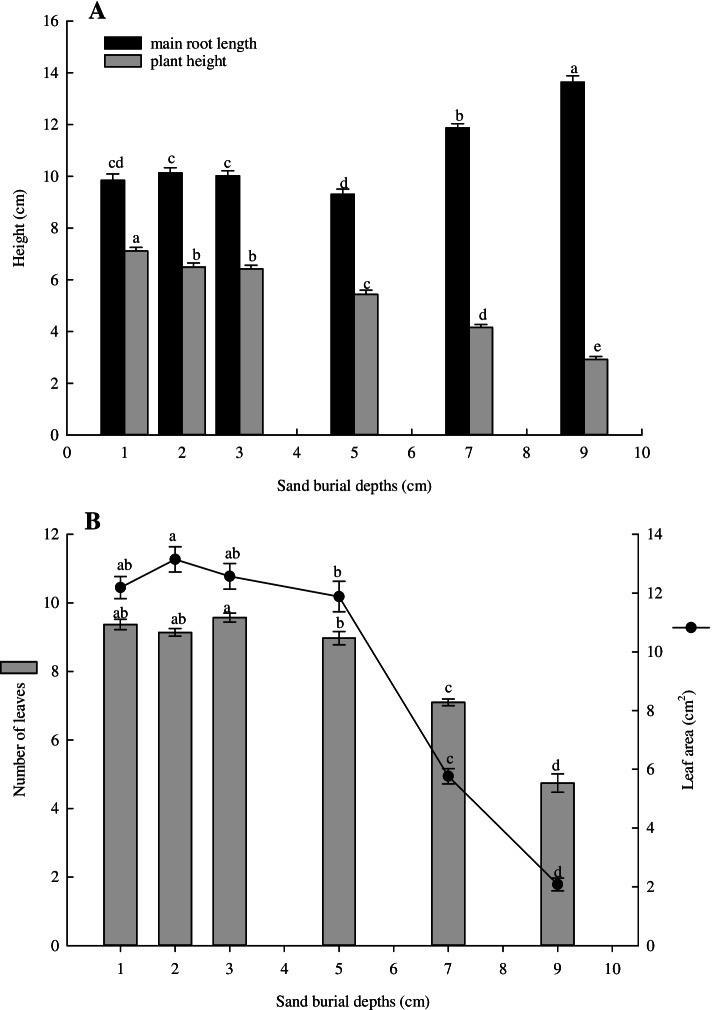
Morphological characteristics of *X. spinosum* seedlings at different sand burial depths. (**A** Main root length and plant height of *X. spinosum* at different sand burial depths; **B** The number of leaves and leaf area of *X. spinosum* at different sand burial depths). Different lowercase letters indicate significant differences among the different sand burial depths of *X. spinosum* by using one-way ANOVA from LSD tests (*P* < 0.05). The values were represented as means ± SE

Figure 3A shows that the main root length and plant height were significantly affected by sand burial depth (main root length, *F* = 48.5, *P* < 0.001; plant height, *F* = 105.5, *P* < 0.001, respectively). The length of the main root at 9-cm burial depth was significantly higher than that in other treatment groups (*P* < 0.001), at 13.6 m, and the minimum length of main root in at 5 cm sand burial depth was 9.3 cm. There was no significant difference in root length at sand burial depths of 1, 2 and 3 cm (*P >* 0.05), with an average value of 10.0 cm for these depths.

As sand burial depth increased, plant height gradually decreased (Fig. 3A). At 1-cm sand burial depth, the plant height was significantly higher than that of other treatment groups (*P* = 0.002), at 7.1 cm, and the minimum plant height at 9 cm was 2.9 cm. There was no significant difference in plant height between 2- and 3-cm sand burial depth (*P* = 0.749).

The number of leaves and leaf area was also significantly affected by sand burial depth (number of leaves, *F* = 97.4, *P* < 0.001; leaf area, *F* = 103.7, *P* < 0.001, respectively) (Fig. 3B). There was no significant difference in leaf number and leaf area at 1-, 2- and 3-cm sand burial depth (*P* > 0.05), but they were significantly higher than those at 5-, 7- and 9-cm sand burial depth (*P* < 0.05). From 5 cm, the number and area of leaves decreased with sand burial depth. At 5 cm sand burial depth, the number of leaves per *X*. spinosum seedling was about 9, and the leaf area was 11.8 cm^2^, but at 9-cm depth, the number of leaves per *X*. spinosum seedling was only about 5, and the leaf area was only 2.1 cm^2^.

### Biomass accumulation of *X. spinosum* seedlings at different sand burial depths

The biomass accumulation of *X. spinosum* seedlings was significantly affected by sand burial depth (root biomass, *F* = 81.0, *P* < 0.001; stem biomass, *F* = 95.8, *P* < 0.001; leaf biomass, *F* = 92.2, *P* < 0.001; reproductive component, *F* = 3.4, *P* = 0.005; total biomass, *F* = 63.0, *P* < 0.001, respectively) (Table 2). With the increase of sand burial depth, the root biomass gradually increased, and the root biomass at 9-cm sand burial depth was significantly higher than that at other sand burial depths (*P* < 0.001) at 0.2854 g, which is about twice of the root biomass at 1-cm sand burial depth. There was no significant difference between the root biomass at 1- and 2-cm sand burial depth (*P* = 0.698), there was significant difference between the root biomass at 2- and 3-cm burial depth(*P* = 0.006), and there was no significant difference between the root biomass at 5- and 7-cm burial depth (*P* = 0.816).

**Table 2:** Biomass accumulation of *X. spinosum* seedlings at different sand burial depths

Different depths(cm)	Root biomass (g)	Stem biomass (g)	Leaf biomass (g)	Reproductive component(g)	Total biomass (g)
1	0.1329 ± 0.0047d	0.1849 ± 0.0055a	0.6109 ± 0.0185a	0.0336 ± 0.0021a	0.9933 ± 0.0258a
2	0.1360 ± 0.0049d	0.1545 ± 0.0058b	0.5341 ± 0.0190b	0.0374 ± 0.0090a	0.8855 ± 0.0300b
3	0.1608 ± 0.0047c	0.1411 ± 0.0055bc	0.5817 ± 0.0202ab	0.0345 ± 0.0017a	0.9459 ± 0.0293ab
5	0.2041 ± 0.0050b	0.1326 ± 0.0068c	0.5338 ± 0.0275b	0.0390 ± 0.0055a	0.9363 ± 0.0379ab
7	0.2060 ± 0.0063b	0.0625 ± 0.0027d	0.2483 ± 0.0103c	0.0173 ± 0.0057b	0.5213 ± 0.0168c
9	0.2854 ± 0.0110a	0.0408 ± 0.0028e	0.1097 ± 0.0121d	0.0000 ± 0.0000c	0.4359 ± 0.0185c

The values were represented as means ± SE Different lowercase letters indicate significant differences among the different sand burial depths of *X. spinosum* by using one-way ANOVA from LSD tests (*P* < 0.05)

As sand burial depth increased, the stem biomass gradually decreased. The stem biomass at 1-cm burial depth was significantly higher than that at other sand burial depths (*P* < 0.001), at 0.1849 g, which was about 4.5 times the stem biomass at 9-cm sand burial depth (0.0408 g).

As sand burial depth increased, the leaf biomass showed a decreasing trend. The leaf biomass (0.6109 g) at 1-cm sand burial depth was significantly higher than that at 9-cm sand burial depth (0.1097 g) (*P* < 0.001). There was no significant difference in leaf biomass at sand burial depths of 1-, 2-, 3- and 5-cm (*P* > 0.05).

There was no significant difference among reproductive components at sand burial depths of 1-, 2-, 3- and 5-cm (*P* > 0.05). At 9-cm sand burial depth, there was no reproductive component.

The total biomass and leaf biomass both showed an overall decreasing trend with sand burial depth. The total biomass (0.9933 g) at 1-cm sand burial depth was significantly higher than that at 9-cm sand burial depth (0.4359 g) (*P* < 0.001), which was about twice as high. There was no significant difference in total biomass at 2-, 3- and 5-cm sand burial depths (*P* > 0.05).

### Biomass distribution of *X. spinosum* seedlings at different sand burial depths

The biomass allocation of *X. spinosum* seedlings was significantly affected by sand burial depth (root biomass ratio, *F* = 387.4, *P* < 0.001; stem biomass ratio, *F* = 60.7, *P* < 0.001; leaf biomass ratio, *F* = 160.1, *P* < 0.001; root to shoot ratio, *F* = 91.6, *P* < 0.001, respectively) (Table 3). With the increase of sand burial depth, the root biomass ratio gradually increased, and the root biomass ratio at 9-cm sand burial depth was significantly higher than that at other sand burial depths (*P* < 0.05). However, as sand burial depth increased, the stem biomass decreased gradually, and the stem biomass at 1 cm sand burial depth was significantly higher than that at other sand burial depths (*P* < 0.05). As sand burial depth increased, the leaf biomass ratio showed a decreasing trend. The biomass ratio of leaves at 1-, 2- and 3-cm sand burial depth was significantly higher than that at 9-cm sand burial depth (*P* < 0.05). The root to shoot ratio increased gradually with the increase of sand burial depth, among which the root to shoot ratio at 9-cm sand burial depth was significantly higher than that at other burial depths (*P* < 0.05), and there was no significant difference between root to shoot ratios at 1-, 2-, 3- and 5-cm sand burial depth (*P* > 0.05).

**Table 3:** Biomass distribution of *X. spinosum* seedlings at different sand burial depths

Sand burial depth (cm)	Root biomass ratio	Stem biomass ratio	Leaf biomass ratio	Root to shoot ratio
1	0.1359± 0.0046e	0.1881 ± 0.0044a	0.6115 ± 0.0078a	0.1600 ± 0.0069c
2	0.1572 ± 0.0050de	0.1764 ± 0.0051b	0.6022 ± 0.0095a	0.1896 ± 0.0076c
3	0.1754 ± 0.0048d	0.1493 ± 0.0031cd	0.6106 ± 0.0062a	0.2160 ± 0.0073c
5	0.2336 ± 0.0073c	0.1406 ± 0.0048d	0.5553 ± 0.0110b	0.3142 ± 0.0133c
7	0.4033 ± 0.0101b	0.1195 ± 0.0031e	0.4693 ± 0.0082c	0.7113 ± 0.0313b
9	0.6753 ± 0.0251a	0.0918 ± 0.0051f	0.2329 ± 0.0212d	3.1712 ± 0.3780a

The values were represented as means ± SE. Different lowercase letters indicate significant differences among the different sand burial depths of *X. spinosum* by using one-way ANOVA from LSD tests (*P* < 0.05)

## Discussion

Seed germination and seedling growth are affected by many environmental factors such as habitat disturbance. Sand burial at different depths change the temperature, moisture and light conditions around the seeds, subsequently affecting seed germination and seedling growth [6, 16, 28, 30]. Sand burial can adjust the emergence time of seeds [24], and can maintain high humidity, thus preventing the seeds directly exposed to the soil surface from being damaged at high temperature or low temperature, increasing the seedling growth speed. The results of the current study showed that sand burial depth had significant effects on the first emergence time, peak emergence time, emergence rate, seedling growth height, biomass and biomass distribution of *X. spinosum* seeds.

As sand burial depth increased, the emergence rate and seedling height gradually decreased. These results are consistent with those of Maun and Riach [12] and Chen et al. [4] It may be that shallow sand burial can protect seeds from the fluctuation of the external environment, and maintain a suitable and stable light, temperature and humid environment for seed germination and seedling growth [1, 13]. In addition, shallow sand burial is more conducive to the rejuvenation of seeds, increasing seed germination rate and seedling emergence speed [20]. In contrast, the deep sand burial environment lacks oxygen, light and warmth, and thus the seed germination activity is reduced [15], and seedling growth is inhibited.

The morphological characteristics of *X. spinosum* seedlings at different depths show that the plant height, the number of leaves and leaf area decreases with the deepening of sand burial depth. In contrast, the root length increases with sand burial depth. This was mainly because under shallow sand, more resources are used for the growth of aboveground parts to ensure normal photosynthesis, but with the increase of sand burial depth, the proportion of resources allocated by seedlings to the aboveground parts decreased. If the seed is buried too deep in the sand, the air permeability of the soil is decreased and the light is weakened, thereby inhibiting seed germination and seedling emergence, while although after germination, the seedlings are inhibited by the exhaustion of energy before unearthing [8], and thus do not continue to grow or even die.

The accumulation and distribution of biomass strongly reflect the growth and development of plants. Different sand burial depths affect the rhizome allocation pattern of seedlings. Research focusing on *Nitraria sphaerocarpa* by Seiwa et al. [14] and *Anabasis aphylla* by Wang et al. [23] showed that with the increase of depth, the proportion of seedlings allocated to stems will gradually increase, whereas the ratio of roots and stems decrease as burial depth increases. However, in the current experiment, the root to shoot ratio gradually increased as sand burial depth increased. This is mainly because the storage in the seeds is the only source of energy, and the nutrients and energy stored in the seeds are limited, so the seedlings consume the nutrients stored in the seeds when they are unearthed. Therefore, when buried in deep sand, the seedlings allocate more biomass for root growth, which can quickly absorb the water in the deep soil and promote the growth of the aboveground parts. This result is consistent with Wang et al. [22], Zuo  et al. [30] and Zhu  et al. [29], who indicated that the biomass allocation trends of different species have different responses to sand burial through their long-term ecological adaptation, and may be related to the unique adaptability of plants to their living environment. Different material collection sites and soil characteristics will also cause changes in physiological and ecological characteristics of plants, resulting in variations in results.

In summary, *X. spinosum* germinated at 1–9 cm sand burial depth, but the emergence speed was fastest and the emergence rate was highest at 1-cm sand burial depth. As sand burial depth increased, the emergence rate gradually decreased. Sand burial depth affects the biomass allocation of *X. spinosum*. As sand burial depth increased, root to shoot ratio increased with the increase of depth, and seedlings allocate more biomass for root growth. Therefore, shallow sand burial is beneficial to the emergence of *X. spinosum* seeds, the growth and morphogenesis of seedlings, and the invasion and spread of this plant in arid areas, whereas deep burial is not conducive to *X. spinosum* invasion. Under the current environmental conditions, it is extremely urgent to strengthen the ecological management of *X. spinosum*. Therefore, suggestions for the prevention and control of *X. spinosum* invasion are as follows. The findings of this study indicate that deep ploughing would be beneficial before sowing crops to ensure that the seeds of *X. spinosum* in the soil are ploughed into deep soil. The results of this study will provide the basis for further study on population reproduction and diffusion mechanism, and the further assessment of prevention, control of *X. spinosum*.

## Methods and materials

Plant seeds were collected in the field and did not need to a collection license, because they are invasive plants and invasive weeds with a wide range of growth. In order to ensure the availability of seeds, they were collected uniformly and identified as *X. spinosum* after screening by teachers. Yu Zhao formally identified the samples.

### Experimental material

The seeds of *X. spinosum* were collected from Huocheng County, Ili Kazak Autonomous Prefecture, Xinjiang in mid-October, 2018. This area belongs to a temperate continental semi-arid climate, with a mild climate, four distinct seasons and abundant sunshine.

A wild *X. spinosum* was selected, and mature involucres (brown, usually containing two seeds) were collected at the end of the *X. spinosum* growth period in October. They were brought to the laboratory, sorted to ensure the full involucre was retained, air-dried naturally for a week and stored in the fridge at 4°C for later use. Before the experiment, the uniform and full involucre seeds were selected, disinfected with 2.0% NaClO sodium hypochlorite solution for 10 min, washed with distilled water three times and then soaked with distilled water for 48 h in order to accelerate germination.

### Research methods

The involucres were sown after the seed soaking treatment. The sand from desert was placed in a perforated pot with double filter paper, and paved. The pot size was 21 cm × 15 cm × 18 cm, and the bottom sand was 9, 8, 7, 5, 3 and 1 cm. Twenty seeds were placed in each pot and then covered with fine river sand with thickness of 1, 2, 3, 5, 7 and 9 cm to obtain the different sand burial treatments. Each treatment group was included 10 pots, and the experiment was repeated three times. The pots were covered with plastic film to prevent evaporation of water in surface river sand. After planting, the pots were placed in an incubator, with temperature set at 28°C during the day (14 h of illumination at 12000 LX) and 15°C at night (10 h). The experiment began in June, 2019, and the emergence was recorded from the surface layer of germ-exposed soil. The emergence number was recorded and marked every day, and three seedlings with the same emergence time and height were selected, and others were removed. The nutrient solution (0.5 times MS medium (Table 4) dissolved in 1 liter of water) was added once every 5 days, 200 mL per basin, and the plant height of the seedlings was measured the next day. The experimental period was 36 days.

**Table 4:** MS medium composition and concentration

	Components	Concentration (mg·L^-1^)
A large number of elements	KNO_3_ NH_4_NO_3_	1900
		1650
	MgSO_4_·7H_2_O	370
	KH_2_PO_4_	170
	CaCl_2_·2H_2_O	440
Iron salt	Na_2_·EDTA	37.25
	FeSO_4_·7H_2_O	27.85
Trace elements.	MnSO_4_·4H_2_O	22.30
	ZnSO_4_·7H_2_O	8.6
	H_3_BO_3_	6.2
	KI	0.83
	Na_2_MoO_4_·2H_2_O	0.25
	CuSO_4_·5H_2_O	0.025
	CoCl_2_·6H_2_O	0.025

### Index determination

The initial emergence time was taken as the date when the germ first emerged from the soil surface after sand burial. The peak time of emergence was taken as the date when the number of seeds emerging was the highest after sand burial.

After sampling, the plant height and root length of seedlings were measured with a ruler, and the fresh weights of roots, stems, leaves and reproductive components (inflorescence and involucre)were weighed with an electronic balance. They were then naturally air-dried for one week, and dry weight was recorded.

Characteristics included main root length (cm), plant height (cm), leaf number, root biomass (g), stem biomass (g), leaf biomass (g) and reproductive components (g) recorded.

The calculated indexes include total biomass (sum of all biomass), root biomass ratio (root weight/total weight), stem biomass ratio (stem weight/total weight), leaf biomass ratio (leaf weight/total weight) and root to shoot ratio (underground biomass/aboveground biomass).

### Statistical methods

SPSS 19.0 (SPSS, Chicago, IL, USA) was used to collate the experimental data and conduct data statistics and analysis. Based on normal distribution of data, one-way ANOVA was used to determine the effects of burial depth on seed germination, and seedling growth. The least significant difference (LSD) method was used for multiple comparisons (*P* < 0.05), and SigmaPlot 14.0 (Systat Software Inc., San Jose, CA) was used to generate figures.

## Data Availability

The datasets generated and/or analysed during the current study are not publicly available due the data is still being supplemented and improved, and it needs to be reflected in the graduation thesis in the later stage, but are available from the corresponding author on reasonable request.
